# Submandibular displacement of a mandibular third molar root during extraction: a case report

**DOI:** 10.1186/1757-1626-3-8

**Published:** 2010-01-06

**Authors:** Kivanc Kamburoglu, Sebnem Kursun, Bengi Oztas

**Affiliations:** 1 Department of Oral Diagnosis and Radiology, Faculty of Dentistry, Ankara University, Ankara, Turkey

## Abstract

A 46-year-old female patient with complaints of pain and swelling on right submandibular region and limitation on mouth opening was reported. She had undergone an unsuccessful surgical procedure under local anesthesia performed by a general practitioner for removal of impacted mandibuler right third molar 1 week earlier. On clinical examination floor of the mouth was tender to palpation. Panoramic and the periapical radiographs showed presence of a radiopaque mass similar to that of a tooth root. Computed tomography scans were obtained for detailed radiographic examination, thereby the presence of a high density area in the submandibular region was detected. Under general anesthesia the displaced root was removed and the postoperative course was uneventful.

## Introduction

The frequency of complications can be expected to increase as the number of surgical extraction operations of impacted mandibuler third molars increases [1]. Displacement of a tooth or a tooth fragment into important adjacent anatomic sites is among other complications that can occur during third molar removal such as; infection, alveolar osteitis, dysesthesia, hemorrhage and anesthetic complication [2]. Although this is a well known complication published case reports are sparse. The most common sites of displacement are the maxillary sinus and the submandibular space [3]. Besides anatomic considerations, such as distolingual angulation of the tooth or dehiscence in lingual cortical plate, excessive or uncontrolled force, improper manipulation and inadequate clinical and radiographic examination are important factors that can lead to tooth displacement [4].

## Case presentation

A 46-year-old Turkish female patient was referred to Ankara University, Faculty of Dentistry, Oral Diagnosis and Radiology Department with complaints of pain, slight swelling on the right side of mouth floor and discomfort during swallowing and limitation in mouth opening. Patient history revealed that a week earlier she had undergone an unsuccessful surgical procedure under local anesthesia performed by a general practitioner for removal of an impacted mandibular third molar. The tooth broke during extraction. The procedure was described by the patient as being difficult and complicated.

**Figure 1:**
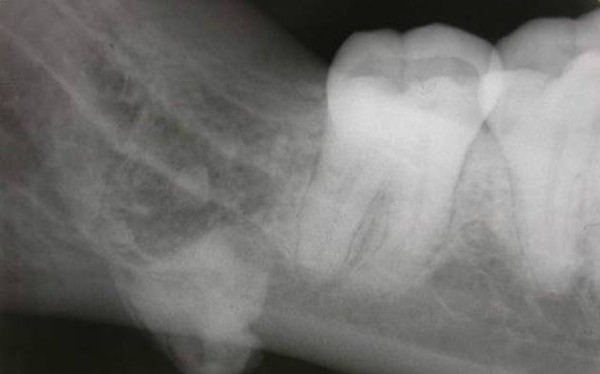
**Intraoral film shows the presence of the tooth root in the submandibular region**.

**Figure 2:**
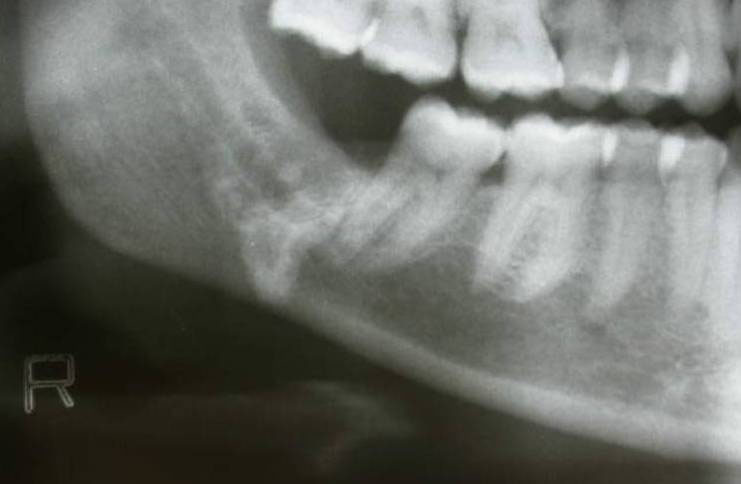
**Panoramic view of displaced mandibuler third molar root**.

**Figure 3:**
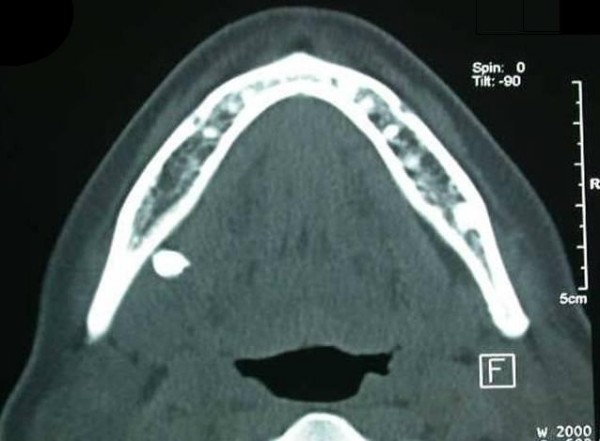
**Axial Computed Tomography scan showing high density area**.

A hard mass was palpated on the posterior region of the mouth floor on clinical examination. Radiographic examination was performed by use of intraoral periapical radiography, panoramic radiography and computed tomography (CT). (Figure 1, Figure 2 and Figure 3). Periapical and panoramic radiography showed the presence of a radiopaque lesion that is similar to the appearance of third molar tooth root. Two dimensional radiographs were inadequate in this case. For detailed radiographic examination, computed tomography scans were taken by spiral technique and continuous 2.5 mm axial sections were obtained. Images were reconstructed to form sagittal and coronal sections and examined. Computed tomography examination demonstrated the presence of a high density area located in the right submandibular region.

**Figure 4:**
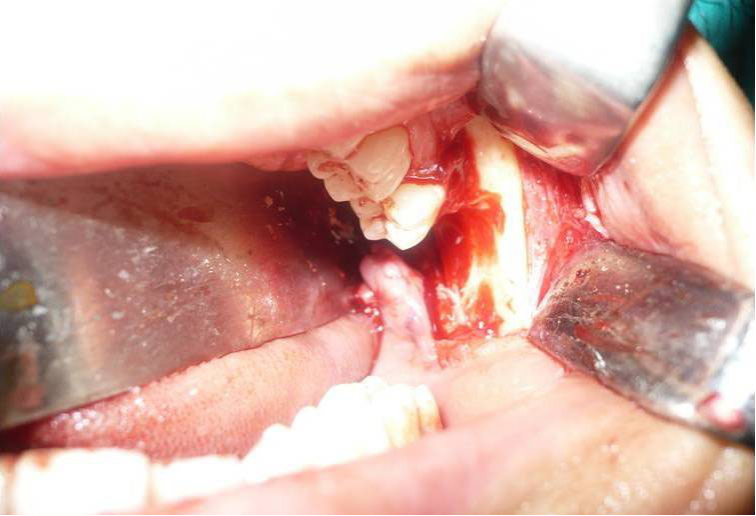
**Intra-operative view of surgical site**.

**Figure 5:**
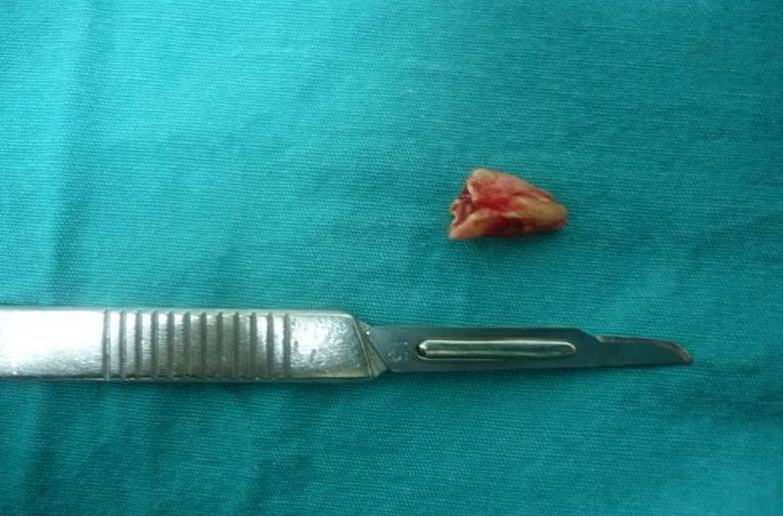
**Root fragment retrieved after surgery**.

Under general anesthesia an incision starting from buccal sulcus towards distobuccal angle of the second molar at gingival margin was extended to the coronoid process directly in line with the anterior surface of ramus. The dislodged root was found by means of blunt dissection and grasped with a pair of artery forceps and removed (Figure. 4, Figure. 5). Patient was given oral antibiotics for 1 week. The postoperative course was uneventful and the patient was asymptomatic at the follow-up visit 3 months later. A written consent was obtained from the patient for case presentation.

## Discussion

It is possible that any tooth fragment lost in the submandibular region could prove difficult to retrieve but it would seem that this is a very rare complication of extraction and can not easily be anticipated. We speculate that in the current case tooth crown broke during extraction and tooth root pushed through the submandibular space by the elevator. Besides, efforts made to retrieve the tooth after its initial dislodgement and blind probing appear to be the possible reasons for further displacement from the submandibular space.

Some authors prefer to postpone surgery for several weeks to allow fibrosis to occur and stabilize the tooth in a firm position. However delayed intervention may increase the risk of infection and result in a foreign body reaction or migration of the tooth [5-7]. Therefore, in the present case surgical operation was performed immediately and the patient was put on a regimen of oral antibiotics for 1 week.

In this case computed tomography was utilized in terms of locating the tooth, assessing the adjacent structures and perforations of the bone and guiding the surgical procedure. It is our belief that obtaining computed tomography scans before retrieval surgery of displaced tooth fragments would be useful.

Adequate clinical and radiographic examination should be performed before third molar removal. The frequency of accidental tooth displacement may be reduced if advanced imaging techniques are often used before surgery.

## Consent

Written informed consent was obtained from the patient for publication of this case report and accompanying images. A copy of the written consent is available for review by the Editor-in-Chief of this journal.

## Competing interests

The authors declare that they have no competing interests.

## Authors' contributions

SK and BO analyzed and interpreted the patient data. KK wrote the manuscript. All authors read and approved the final manuscript.
